# Neuroendocrine neoplasms of the pancreas: diagnosis and pitfalls

**DOI:** 10.1007/s00428-021-03211-5

**Published:** 2021-10-13

**Authors:** Björn Konukiewitz, Moritz Jesinghaus, Atsuko Kasajima, Günter Klöppel

**Affiliations:** 1 Institute of Pathology, Universitätsklinikum Schleswig-Holstein, Campus Kiel, Christian-Albrechts-Universität zu Kiel, Arnold-Heller-Straße 3/14, 24105 Kiel, Germany; 2 Institute of Pathology, Universitätsklinikum Marburg, Baldingerstraße, 35043 Marburg, Germany; 3 Institute of Pathology, Technische Universität München, Trogerstraße 18, 81675 Munich, Germany

**Keywords:** Pancreatic neuroendocrine neoplasms, Diagnosis, Histology, Immunohistology, Pitfalls

## Abstract

Common to neuroendocrine neoplasms of the pancreas is their expression of synaptophysin, chromogranin A, and/or INSM1. They differ, however, in their histological differentiation and molecular profile. Three groups can be distinguished: well-differentiated neuroendocrine neoplasms (neuroendocrine tumors), poorly differentiated neuroendocrine neoplasms (neuroendocrine carcinomas), and mixed neuroendocrine-non-neuroendocrine neoplasms. However, the expression of synaptophysin and, to a lesser extent, also chromogranin A is not restricted to the neuroendocrine neoplasms, but may also be in a subset of non-neuroendocrine epithelial and non-epithelial neoplasms. This review provides the essential criteria for the diagnosis of pancreatic neuroendocrine neoplasms including diagnostic clues for the distinction of high-grade neuroendocrine tumors from neuroendocrine carcinomas and an algorithm avoiding diagnostic pitfalls in the delineation of non-neuroendocrine neoplasms with neuroendocrine features from pancreatic neuroendocrine neoplasms.

## Introduction

Diagnosis and pitfall are like two sides of the same coin. The better the diagnostic criteria, the less the number of diagnostic pitfalls. However, careful processing of diagnostic failures has helped in many cases to improve diagnostic criteria. In this article on pancreatic neuroendocrine neoplasms (PanNENs), we will therefore focus on both, presenting the most important diagnostic criteria and providing clues to avoid main pitfalls.

PanNENs belong to the tumors that bear the generic name “neuroendocrine neoplasms.” This name is used as a collective term for two tumor families that share the expression of neuroendocrine markers, such as synaptophysin and chromogranin A, but differ distinctly in their morphological and molecular profiles [1, 2]. In the first group, growth and behavior is slower and individually more different than in the second group, where it is generally faster [3]. Both groups of NENs can arise almost anywhere in the body, even though they all show a strong preference for the gastroenteropancreatic system and the lung, with a varying and interesting site-specific distribution. For historical reasons, the world health organization (WHO) classifications of the NENs of the various organ systems do not follow a uniform terminology [4]. However, the WHO generally follows the principle of distinguishing between well and poorly differentiated NENs and the delimitation of mixed neoplasms [1, 2, 5–7].

The PanNENs play a pioneering role in the classification of NENs because they are frequent among the NENs, have a very varied morphology, and may show a multifaceted functionality [1]. Currently, they are represented in two WHO “blue books,” the classifications of tumors of endocrine organs and the classification of digestive system tumors [1, 2]. This review aims to outline the morpho-genetic characteristics of pancreatic NENs and to provide a practical approach to daily routine diagnostics with highlighting of main diagnostic pitfalls and important NEN mimics.

## Diagnostic features

The 2017 and 2019 WHO classifications stratify the PanNENs into well-differentiated NENs (pancreatic neuroendocrine tumors, PanNETs) and poorly differentiated NENs (pancreatic neuroendocrine carcinomas, PanNECs) and presume that all PanNENs have a malignant potential, however, with a different probability to metastasize [1, 2].

### PanNET

**Fig. 1:**
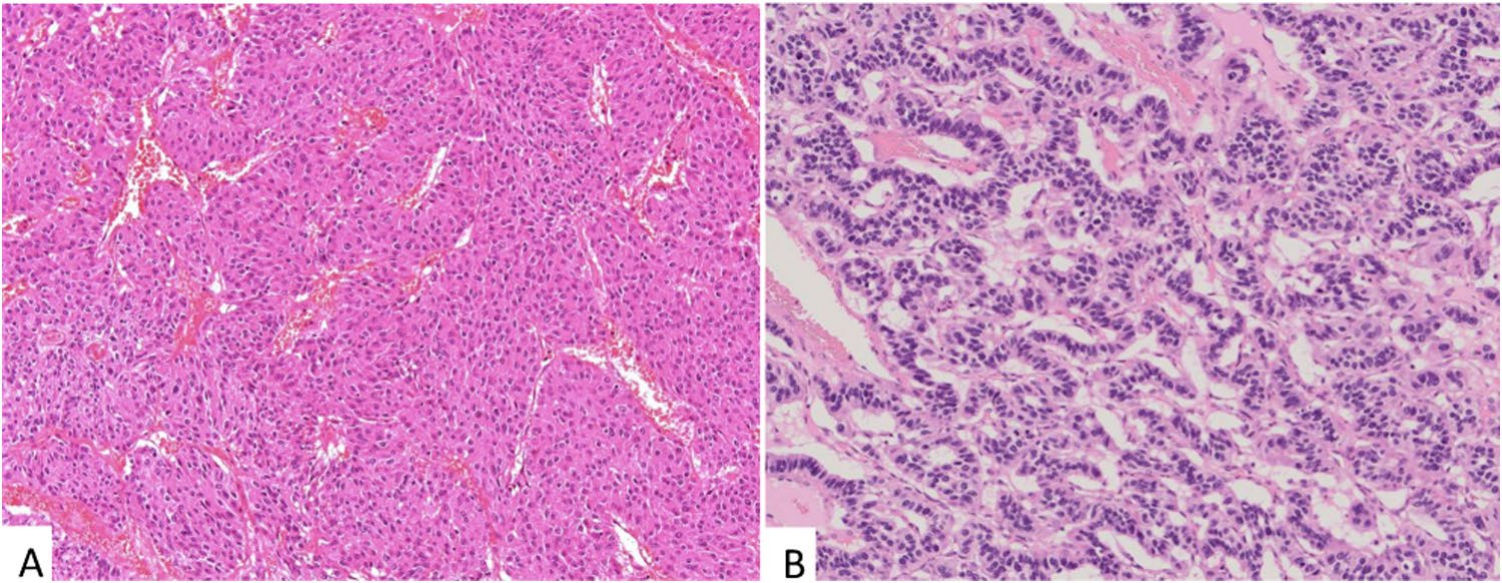
Neuroendocrine tumors with solid (**A**) and trabecular (**B**) growth patterns

**Fig. 2:**
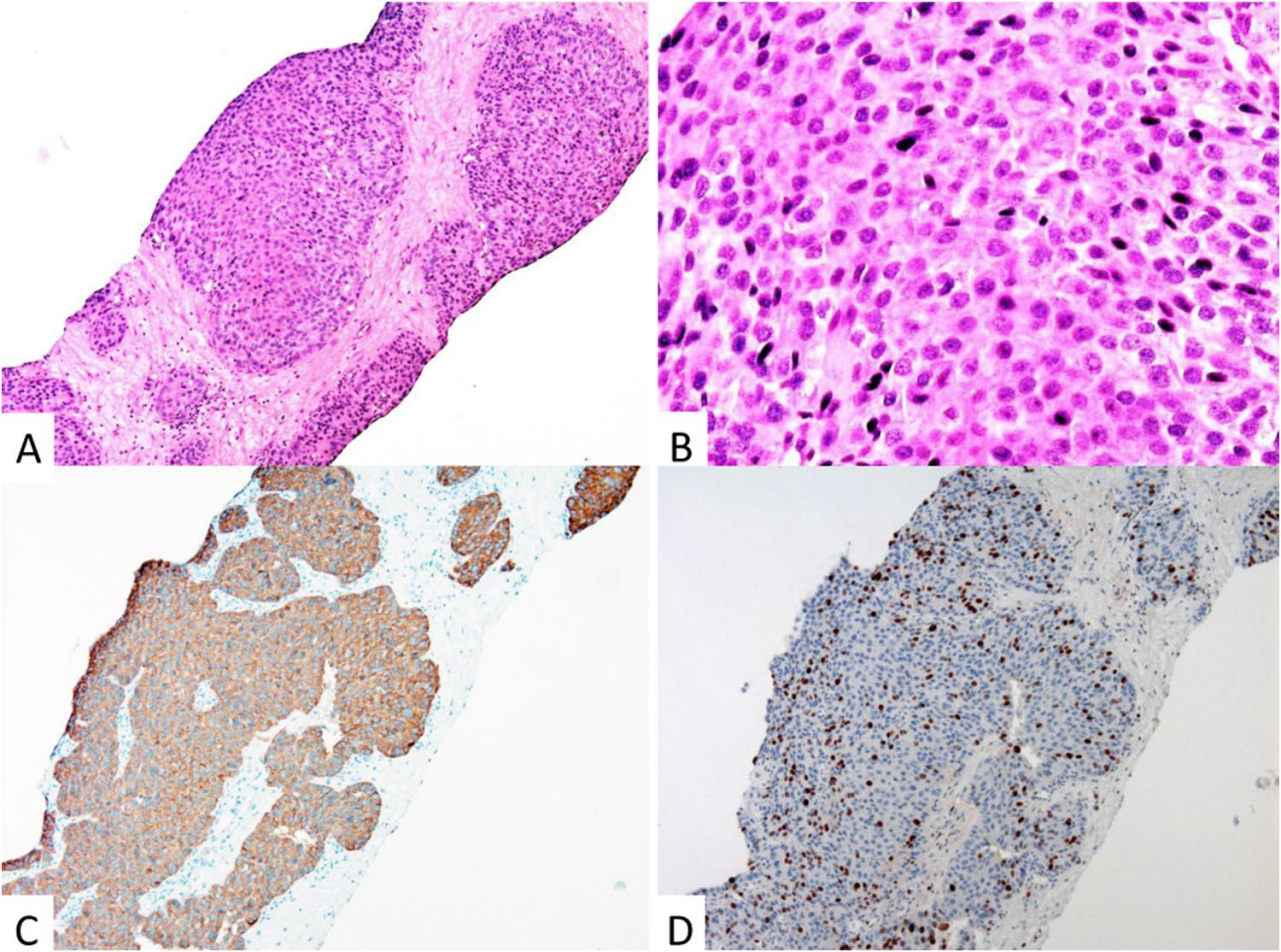
Liver metastasis of a neuroendocrine tumor G3 with an organoid growth pattern (**A**) showing monomorphous and round nuclei (**B**) and expression of synaptophysin (**C**) and Ki67 (index 25%) (**D**)

The *histological profile* of PanNETs is characterized by an organoid growth pattern with an ordered arrangement of cells, a variable amount of fibrotic stroma, and only rare necrotic changes (Figs. 1 and 2). Although the organoid pattern is not uniform but very diverse, the histological diversity can probably be traced back to either a solid or trabecular architecture that can be subdivided due to the composition of the stroma and its vascularization into solid-nested, solid-paraganglioma-like, solid-microglandular, and trabecular-reticulated, trabecular-gyriform, and trabecular-cystic patterns [8–11]. Interestingly, some of these patterns seem to have a relationship to the functionality of the tumor cells, because they show a strong association with the expression of certain hormones (see below).

The cells of this organized tumor tissue mostly display an eosinophilic cytoplasm and mainly round uniform nuclei with hyperchromatic (pepper and salt) chromatin, small inconspicuous nucleoli, and a rather low mitotic rate. PanNETs with oncocytic, lipid-rich (clear), or hepatoid cells or with pleomorphic nuclei are rare [11]. PanNETs are usually well demarcated from the surrounding parenchyma, when they are small (< 1 cm). When they are larger, they can widely infiltrate into the adjacent acinar tissue, thereby invading vessels and nerves and entrapping preexistent islets or single ducts. Rare PanNETs show a peculiar mixture of solid or trabecular cell clusters with small non-neoplastic ducts often embedded in the sclerotic stroma [1–3, 12, 13].

**Fig. 3:**
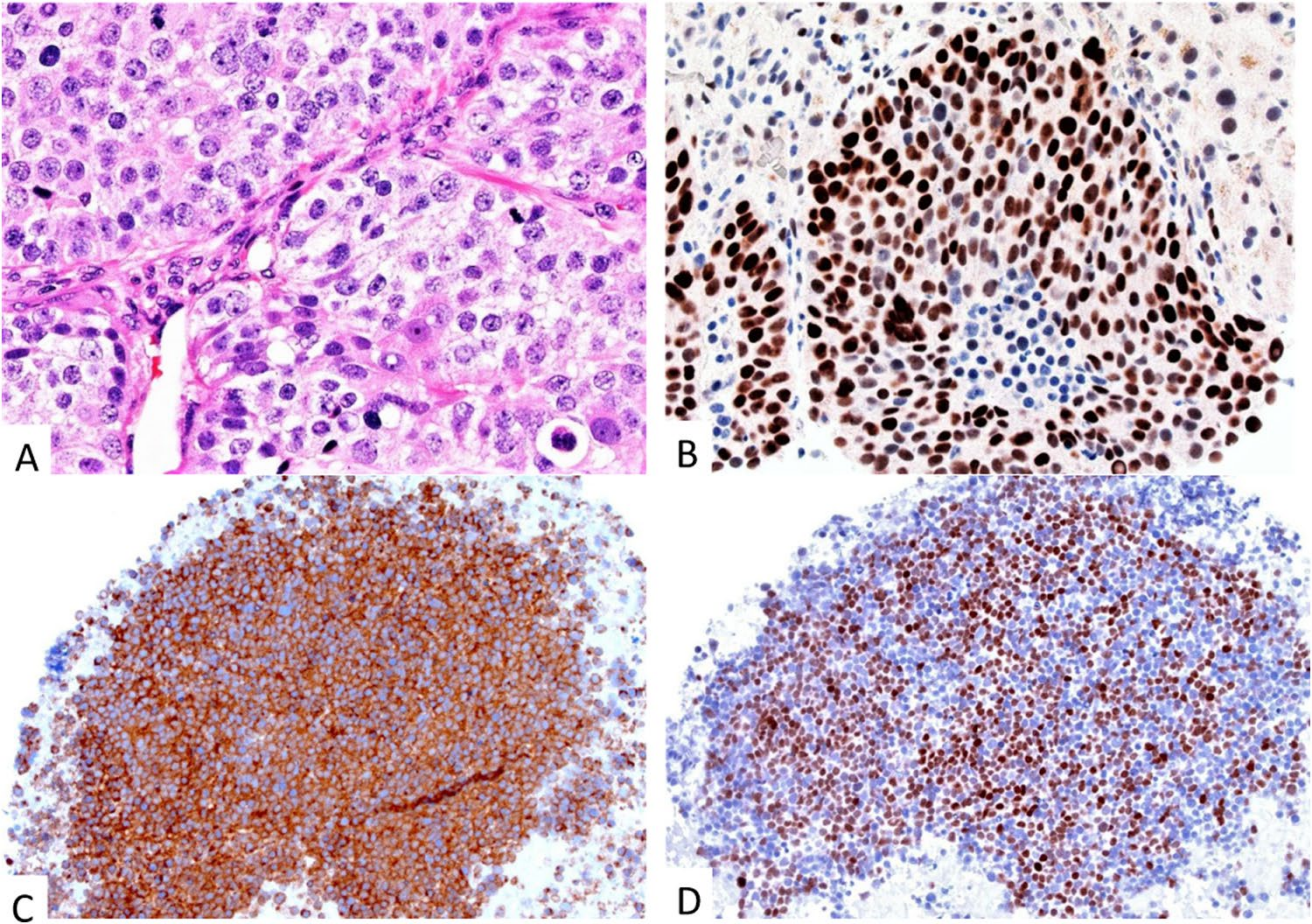
Neuroendocrine carcinoma, large cell type: solid cell clusters with pleomorphic nuclei showing a prominent nucleolus (**A**), and an overexpression of p53 (**B**). Fine-needle aspiration cytology specimens with matching expression of synaptophysin (**C**) and INSM1 (**D**)

The *immunohistochemical* profile of PanNETs that is essential to establish the diagnosis includes the expression of cytokeratin, synaptophysin, and chromogranin A and Ki67 (Fig. 2) [14]. In the case of a PanNET, G3 staining for p53 and RB1 is highly recommended to distinguish these tumors from PanNECs (Fig. 3). The staining of peptide hormones, of the somatostatin receptor 2A (SST2) or the site-specific transcription factor ISLET-1, is recommended where the diagnosis needs it to be complete [4, 8, 15, 16].

Labeling for cytokeratin proves the epithelial nature of a NEN in cases where a neuroectodermal tumor such as a paraganglioma must be excluded [10]. Diffuse and intense cytoplasmic expression of synaptophysin and chromogranin A and nuclear staining for insulinoma-associated 1 (INSM1) (Fig. 3) [17] reveals the tumor’s neuroendocrine differentiation, the common denominator of NENs [18]. The labeling of the nuclei with Ki67 is the best way to accurately determine the proliferative activity of the tumor cells, and this method has largely replaced the counting of mitoses. The exact assessment of the proportion of Ki67-labeled cells as the basis for the calculation of the Ki67 index has emerged as indispensable for the prognostic and therapeutic stratification of PanNETs [19, 20]. The stratification is based on a three-tired grading that separates G1, G2, and G3 PanNETs according to their Ki67 index (Table 1) [1, 2]. PanNETs G3, which represent a new category among NENs, have no defined upper mitotic or Ki67 rate limit; however, usually their mitotic rate and Ki67 index do not exceed 20/10 HPF and 50% (Fig. 2), respectively. Most PanNETs G3 appear to develop from a low-grade NET, since they often manifest themselves as metastases in patients with a prior history of a G1 or G2 PanNET [21].

**Table 1:** Grading of pancreatic neuroendocrine neoplasms (adapted from (1))

	Morphology	Grade	Ki67 index*	Mitosis**
NET	Well differentiated	G1	< 3%	< 2
		G2	3–20%	2–20
		G3	> 20%	> 20
NEC	Poorly differentiated		> 20%	> 20

*Abbreviations*: *NET* neuroendocrine tumor, *NEC* neuroendocrine carcinoma

^*^Counted in at least 500 cells in hot spot areas, **in 2 mm^2^

PanNETs produce peptide hormones which are orthotopic (insulin, glucagon, somatostatin, pancreatic polypeptide, and serotonin) or ectopic (gastrin, vasoactive intestinal polypeptide; VIP, adrenocorticotropin; ACTH; and others) to the pancreas and can be identified by specific antibodies. Approximately 30% of PanNETs are functioning, meaning that the peptide hormone which is produced and secreted also causes a hormonal syndrome. The functioning NETs of the pancreas include insulinoma, glucagonoma, gastrinoma, VIPoma, GRHoma, ACTHoma, or PanNET with carcinoid syndrome and serotonin expression [1, 2]. Whether PanNETs producing and secreting somatostatin can cause a somatostatin syndrome, as described in 1979, is currently under debate, since a study from 2008 was unable to find any syndrome in somatostatin-positive PanNETs or duodenal NETs, and the evidence given in the most recent study is inconclusive [22, 23]. Rarely, there are PanNETs with hypercalcemia, which may produce calcitonin, but the occurrence of hypercalcemia is not necessarily tied to calcitonin secretion [2, 24]. The most frequent functioning PanNETs are insulinomas, which in 90% are small (< 2 cm) and behave benignly [25]. All the other functioning PanNETs are rare and usually show a malignant behavior, especially the tumors with ACTH production and Cushing syndrome. All PanNETs that produce and also secrete a hormone, but are non-syndromic, fall into the category of non-functioning PanNETs and account for about 70% of all PanNETs. Forty percent of PanNETs are multihormonal and are generally found among the non-functioning tumors [2]. Interestingly, the rare (about 20%) malignant insulinomas seem to start in the pancreas as non-functioning multihormonal tumors, with only single insulin cells, but become syndromic after large liver metastases have developed in which the number of insulin-secreting cells is sufficient to produce a hypoglycemic syndrome (GK, personal observation).

It is increasingly noticed that the production of some hormones is associated with certain histological patterns of the PanNETs. Thus, a solid-nested pattern with amyloid is usually associated with the expression of insulin and is found in insulinomas [1]. Tumors with a trabecular-reticulated and often cystic pattern express glucagon [9]. Tumors with a solid paraganglioma-like or solid-microglandular pattern with psammoma-bodies usually contain somatostatin-positive cells [10], and a trabecular sclerosing pattern of a tumor adjacent to the main duct commonly associates with serotonin positive cells [26]. In some multihormonal PanNETs with a clear separation of a solid from a trabecular pattern, each pattern may have its own hormone production.

Membranous SST2 expression on tumor cells is needed to visualize and treat the tumors with radioisotope-labeled somatostatin [16]. It can be detected in most PanNETs, except for insulinomas which express SST2 in only 50% of the tumors and rather express GLP1R than SST2 [27]. If a primary tumor is SST2-positive, it can be assumed that later metastases are also positive and are therefore detectable in the follow-up. Very helpful for the localization of a primary in the pancreas (or duodenum) in case of a liver metastasis with unknown primary is the nuclear expression of the transcription factor ISLET-1 [4, 8, 28].

The *molecular* profile of PanNET is profoundly different from that of pancreatic ductal adenocarcinomas (PDAC). Key drivers of PanNETs are alterations in *MEN1* and *ATRX* or *DAXX* [29–33], while abnormalities of *KRAS, TP53*, *CDKN2A,* and *SMAD4* are the drivers in PDACs [34–36]. *MEN1* is a tumor suppressor gene located on chromosome 11 [37] encoding for the protein Menin, which is an important factor for the regulation of chromatin remodeling [38–40] and seems to play a key role in tumor initiation as *MEN1* alterations are already detectable in pancreatic microtumors [41] and as germline mutation in the genetic syndrome multiple endocrine neoplasia type 1 (MEN1, see below). *MEN1* is known to interact with genes of chromatin modifications, altered telomere length, DNA damage repair, and mTOR signaling, which are the four main genetic pathways involved in the development of pancreatic NETs [42]. *ATRX* and *DAXX* are also genes involved in chromatin remodeling with a high frequency of alterations (40%) in PanNETs [29]. Inactivating mutations of *ATRX* or *DAXX* are associated with an alternative lengthening of telomeres (ALT), a telomerase-independent telomere maintenance mechanism [30]. *ATRX/DAXX* alterations seem to be late events in tumorigenesis as they are only detectable in large fully developed NET [31, 32] but not in microadenomas [41]. Furthermore, NET with *ATRX/DAXX* mutations appear to be associated with a poor prognosis compared to *ATRX/DAXX* wildtype tumors [31, 32, 42]. *ATRX* and *DAXX* are genes that are strongly involved in different epigenetic mechanisms regulating gene expressions per methylation pattern [43]. *MEN1*, *ATRX*, and *DAXX* alterations are associated with a pathological protein expression which is detectable by immunohistochemistry [29, 44]

The third relevant cluster of commonly mutated genes are alterations in genes belonging to the mTOR pathway, which are mutated in about 15% of the pancreatic NET [29, 42], mostly affecting *PTEN*, *TSC1*, and *TSC2* [42]. Less recurrent mutations detected in pancreatic NET involve *ATM*, *YY1*, and *MUTYH* [42, 45, 46]. *TP53* and *RB1* are usually wild types in PanNET, in contrast to PanNEC [15, 16, 47]. A recent study focusing on the DNA-methylation profile distinguished between alpha-like, beta-like, and intermediate PanNET clusters that differed in prognosis [48, 49].

Most NETs are sporadic and solitary tumors. However, approximately 10% of pancreatic NETs develop in association with genetic syndromes and then often manifest as multiple tumors, usually also affecting extrapancreatic organs. The most common syndrome is MEN 1 (see above), followed by the syndromes of Von-Hippel-Lindau, neurofibromatosis type 1, and tuberous sclerosis, with germline mutations in the genes *MEN1*, *VHL*, *NF1*, and *TSC2*, respectively. Functioning PanNETs occur predominantly in MEN1, in which they account for about 30% of the cases and include mainly insulinomas and duodenal gastrinomas.

### PanNEC

PanNECs are rare high-grade pancreatic neoplasms accounting for a maximum of 10% of PanNENs. They arise as sporadic, solitary, and non-functioning neoplasms, and have not been observed in association with genetic syndromes [1, 2]. An association with smoking can be suspected, has so far however not been established.

The *histological profile* of PanNECs is characterized by diffuse solid sheet-like and/or a more irregularly nested pattern (Fig. 3). Common to both patterns are geographical necrosis. NECs with diffuse sheet-like patterns are often composed of highly atypical small- to medium-sized cells that have a scant cytoplasm and hyperchromatic nuclei with inconspicuous nucleoli and focal nuclear molding. NECs with more nested patterns are mostly composed of larger cells whose cytoplasm is rather well-developed and eosinophilic, carrying a polymorphous nucleus with a prominent nucleolus within vesicular chromatin delimited by a delicate nuclear membrane. Mitoses, including atypical mitoses, are common. PanNECs are usually indistinguishable from NECs of other sites by histology alone [1, 2, 4, 16].

The *immunohistochemical* profile of PanNECs that is essential to establish the diagnosis includes the expression of cytokeratin (CK), synaptophysin, INSM1 (Fig. 3), chromogranin A, and Ki67, as well as the overexpression/loss of p53 (Fig. 3) and the loss of nuclear RB1 staining [1, 2, 15, 16].

PanNECs express CK8 and 18. In small cell type PanNECs, CK labeling may show a punctuate pattern, and in exceptional cases, CK labeling can even be lacking. A few PanNECs also express vimentin. Synaptophysin is typically diffusely but faintly and somewhat patchy expressed, often displaying a dot-like pattern. Chromogranin A is usually focally and scarcely expressed and may even be lacking, since neurosecretory granules, whose membranes contain chromogranin A, are rare in NEC cells. CD56 labels the membranes of PanNECs broadly, but it should be never the only neuroendocrine marker on which the diagnosis of a NEC is based, since it has a high degree of unspecificity. PanNECs are mainly ISLET-1-negative [1–3, 8, 12, 15, 16, 50, 51]. All PanNECs show a Ki67 index greater than 20%, with a mean of 50 to 60% (Table 1).

The *molecular* profile of PanNECs is characterized by *TP53* and *RB1* mutations which are the key drivers of PanNECs as well as of extrapancreatic NECs [4, 15, 16, 47, 52–54]. Later studies additionally identified *KRAS* as a third driver in PanNEC [55], suggesting a potential relationship to PDAC. Next-generation sequencing studies using larger gene panels revealed no further recurrent gene mutations in PanNECs and no clear molecular differences between small and large cell subtypes [15]. However, NECs seem to possess an organ-specific signature, since PanNECs have only *KRAS* mutations, while colorectal NECs have also *APC* mutations [15, 52].

*TP53* and *RB1* mutations are important in the distinction of PanNEC from PanNET, as they are absent in G1/G2 PanNETs and are only occasionally present in PanNETs G3 [15, 16, 42]. Immunohistochemically, almost 70% of PanNECs overexpress p53 that reflects an underlying *TP53* alteration, and show loss of RB1 nuclear staining, indicating a *RB1* alteration [15, 16]. Unlike well-differentiated PanNETs, PanNECs retain the expression of DAXX/ATRX, since the corresponding genes are not mutated. SST2 expression is negative in 85% of the tumors [16]. These tumors, which are negative on somatostatin radio receptor scintigraphy, are often positive on FDG-PET [56].

### Mixed neuroendocrine-non-neuroendocrine neoplasms (MiNEN)

PanNENs may contain coexisting high-grade PDAC or acinar cell carcinoma. If one component exceeds 30% (an arbitrarily chosen threshold) of the total tumor cell population, such tumors are called “mixed neuroendocrine-non-neuroendocrine neoplasms (MiNENs).” If the non-neuroendocrine component is an adenocarcinoma and the neuroendocrine component presents as NEC, the old term “mixed adenoneuroendocrine neoplasm (MANEC)” can be retained [1, 2, 57]. In a small series of pancreatic MiNENs, the neuroendocrine as well as the non-neuroendocrine component displayed poor differentiation and either a mosaic or a composite/amphicrine pattern. Single cases of published pancreatic MiNEN revealed a close relationship to PDAC [15], as it was also found in colorectal MiNEN [52, 58], and interestingly also to its precursors, as two cases of pancreatic intraductal papillary mucinous neoplasms (IPMN) associated with NEN were reported, in which the NEN component showed *GNAS* mutations, typical for IPMN. In one case, the NEN component was a NEC [15], and in the other case, a NET [59]. In mixed acinar-neuroendocine carcinomas, the expression of trypsin and synaptophysin can be so intense and overlapping that an amphicrine pattern can be observed. Genetically and biologically, these neoplasms are closely related to the conventional acinar cell carcinomas [60, 61].

## Diagnostic pitfalls

**Fig. 4:**
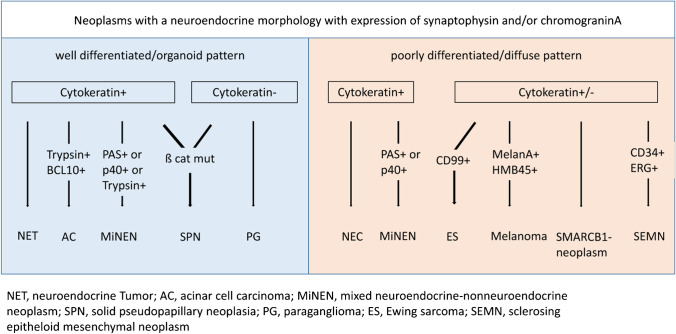
Diagnostic algorithm for the differential diagnosis of synaptophysin expressing pancreatic neoplasms

Pitfalls in the diagnosis of PanNENs concern mainly the confusion of NECs with NETs G3 and of NENs with a variety of non-NENs such as acinar cell carcinoma, solid pseudopapillary neoplasm, pancreatic paragangliomas, PDACs with neuroendocrine cells, and subsets of non-NENs of epithelial and mesenchymal origin with neuroendocrine features (Fig. 4) as well as tumor-like lesions [21, 62].

### PanNET G3 versus NEC

The delimitation between NETs and NECs is important as their clinical management differs fundamentally [63]. In small biopsies and occasionally in resection specimens, the distinction between NETs G3 (also called high-grade NETs) and NECs, especially of large cell type, can be difficult.

Histologically, NETs G3 mostly show an organoid solid or solid-trabecular (rarely pseudoglandular) pattern that is well distinguishable from the usually sheet-like architecture of small cell NECs, but difficult to distinguish from the nested architecture of most large-cell NECs [15, 16, 64, 65], particularly in biopsies. Hyalinized versus desmoplastic stroma and regular versus random vessel pattern are also criteria for the distinction of NET from NEC, but are usually not helpful in biopsies. Helpful are cytological criteria, with more polymorphous nuclei with conspicuous nucleoli in NECs, and immunohistochemical markers, including SST2, p53, and RB1. The vast majority of NETs express SST2 and show a normal (weak and no more than 20%) expression of p53 and RB1 (no complete loss) [16, 64, 65]. In contrast, only 16% of NEC express SST2 and show an abnormal expression of p53 and/or RB1 in 70% of the cases [16]. If the distinction is still difficult, the testing for the expression of hormones can help, as most NETs, but no NECs, are hormone-producing tumors [10].

### Acinar cell carcinoma versus PanNET G3

**Fig. 5:**
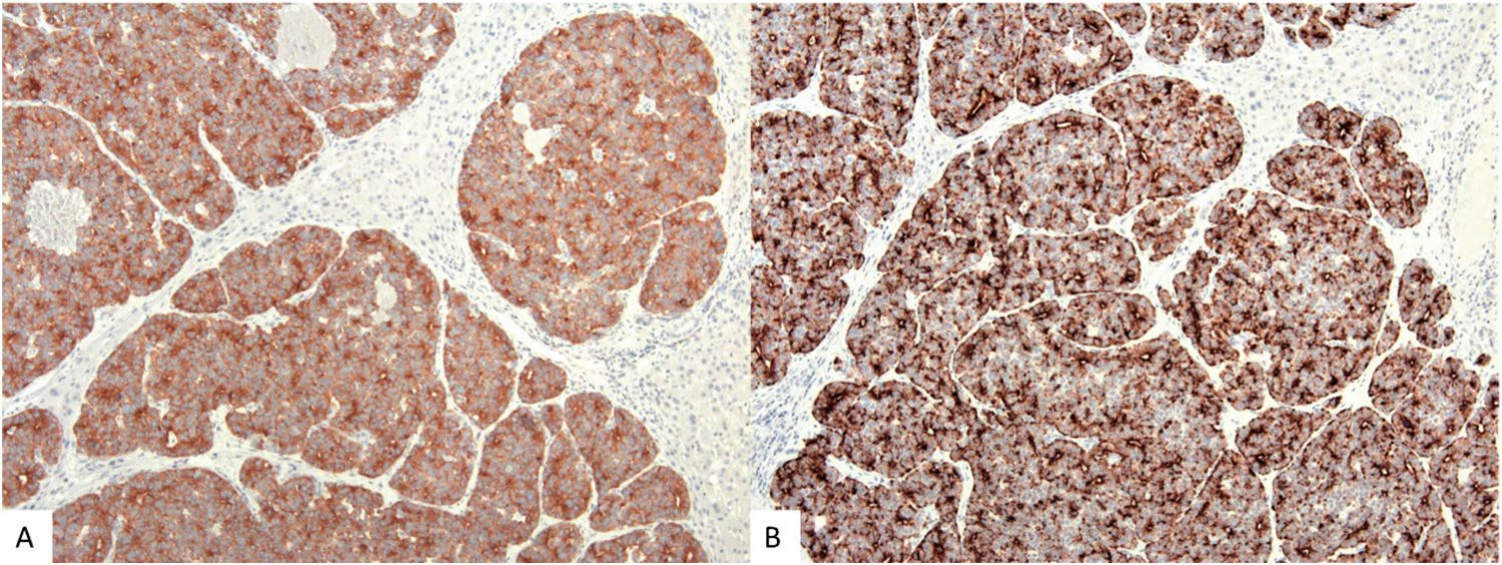
Mixed acinar-neuroendocrine carcinoma with diffuse expression of synaptophysin (**A**) and trypsin (**B**)

Acinar cell carcinomas are histologically recognized by their more or less striking acinar pattern, faint PAS positivity, round nuclei with prominent nucleoli, high mitotic rate, and scant fibrous stroma. However, some tumors displaying solid or trabecular patterns are very reminiscent of NETs [1]. In addition, approximately 40% of acinar cell carcinomas express synaptophysin and chromogranin A, and some of these qualify as mixed acinar-neuroendocrine (ductal) carcinomas, with an intimate and amphicrine mixture of the two components. In the latter tumors, the co-expression of trypsin and synaptophysin can reach such an extent in all tumor cells that, if one only stains for synaptophysin, the tumor, which labels diffusely for synaptophysin, is easily misdiagnosed as NETs (especially NETs G3 when the mitotic activity is high) and only correctly recognized as mixed acinar-neuroendocrine carcinoma when trypsin (and/or BCL10) is added to the marker panel (Fig. 5) [66–70]. Very helpful in these cases are SST2 and ISLET-1, since both markers are negative in acinar cell carcinomas with neuroendocrine features (authors’ personal observation).

### Solid pseudopapillary neoplasm versus PanNET

Solid pseudopapillary neoplasms may present with a predominantly solid and monomorphous cell pattern and the expression of synaptophysin and cytokeratin. These cases, which very much mimic a NET, however, differ from NETs by a lack of chromogranin A staining and a positive nuclear (and cytoplasmic) labeling for ß-catenin, indicating a *CTNNB1*-mutation found in almost all SPN [71–74]. Furthermore, it is helpful that the synaptophysin and cytokeratin staining in SPN is rarely diffuse, but usually patchy [62].

### Paraganglioma-like PanNET versus true paraganglioma

A small fraction of PanNETs shows a solid, paraganglioma-like histology and may therefore mimic rare paragangliomas occurring in or, more commonly, at the pancreas [10]. While paragangliomas usually show a benign behavior, paraganglioma-like PanNETs do not differ biologically from the other PanNETs. The key markers for the distinction of the two entities are CKs [2, 10] and GATA3 [75, 76]. GATA3 is positive in paragangliomas and negative in PanNETs, while CKs are negative in paragangliomas and positive in PanNETs. Furthermore, paragangliomas do not infiltrate into the pancreatic tissue, while paraganglioma-like PanNETs usually do [10, 23].

### Ductal adenocarcinoma with islet cells versus MiNEN

The distinction of conventional PDAC intimately associated with islet cells from MiNEN is in most cases not a problem. First, the WHO classification requires that the neuroendocrine component of MiNENs exceeds 30% of the tumor cell population [1]. In PDAC, the number of islet cells that combine with duct-like glands of a PDAC to form ductal-neuroendocrine complexes is less than 30% of the tumor cell population. In addition, they show no Ki67 labeling as a sign of proliferation in contrast to PDAC cells. Moreover, immunostains for pancreatic hormones identify the cells that associated with PDAC structures as islet cell types. Finally, the metastases of these PDACs never contain neuroendocrine cells of the type found in the pancreas [77].

### Mesenchymal and non-epithelial neoplasms versus NEC

**Fig. 6:**
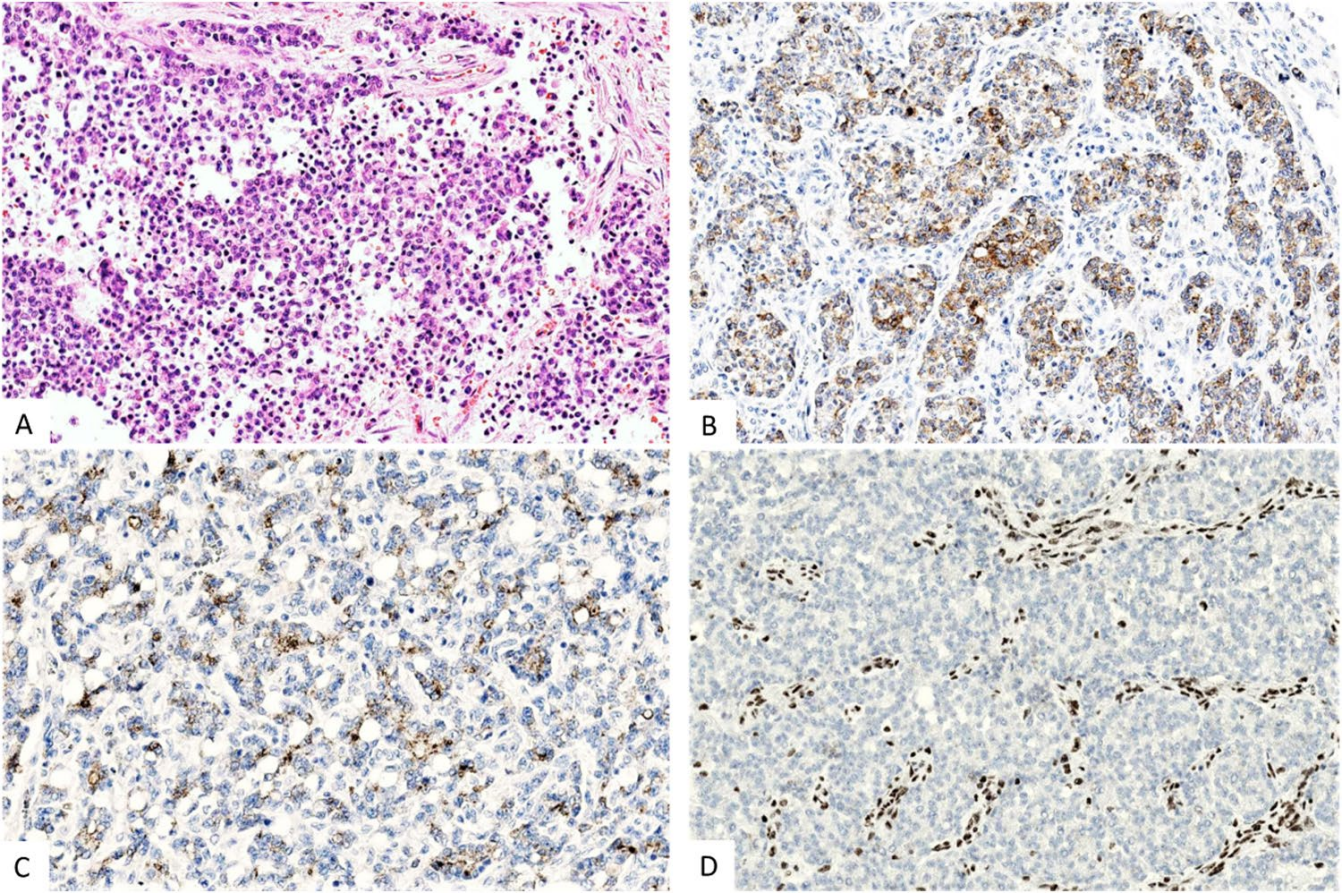
Pancreatic SMARCB1 deficient rhabdoid neoplasm (**A**) with expression of synaptophysin (**B**), chromogranin A (**C**), and expression loss of INI1 (**D**)

Recently a range of mesenchymal and non-epithelial mimickers of neuroendocrine neoplasms, mainly of the large-cell NECs, have been described in a large consultation series [62]. Of particular interest and rather new among these NEN mimickers are tumors from the Ewing Sarcomas group, desmoplastic small round cell tumors, epithelioid neoplasms with *FUS-CREM* gene fusions, epithelioid sarcomas, synovial sarcomas, SMARCA4- and SMARCB1-deficient neoplasms (Fig. 6), clear cell sarcomas of the gastrointestinal tract, alveolar soft part sarcomas, solitary fibrous tumors, chordomas, melanomas, and sclerosing epithelioid mesenchymal neoplasms. Six of these neoplasms were located in the pancreas and included Ewing sarcoma, SMARCB1(INI1)-deficient neoplasms (Fig. 6), melanoma, and sclerosing epithelioid sclerosing neoplasms [62]. To unmask these neoplasms as NEN mimickers, the testing of the key markers CD99, INI1, and S100 is necessary in cases of Ewing sarcomas, SMARCB1-deficient neoplasms, and melanomas, respectively. The sclerosing epithelioid mesenchymal neoplasm of the pancreas is an exceedingly rare pancreatic tumor that has recently been proposed as a new tumor entity with only single reported cases in the literature [78]. The synaptophysin expression in mesenchymal/non-epithelial NEN mimickers is mostly patchy with co-expression of chromogranin A in one-third of the cases [62].

### Tumor-like lesions

Tumor-like lesions as NEN mimickers are islet cell aggregates in specimens of chronic pancreatitis, particularly of the obstructive type of pancreatitis associated with duct occluding tumors. The islet clusters that are found in fibrotic and/or lipomatous tissue devoid of acinar cells can histologically imitate an infiltrating PanNET. To avoid a diagnostic pitfall, the islet cell nature of the cell aggregates has to be demonstrated. This is easily done by immunostainings for insulin and glucagon, which reveal the normal non-random distribution of the two islet types in the islet cell clusters that profoundly differs from the monohormonal expression in most NETs [62]. The same applies to the PP-rich islet clusters in the posterior-caudal and uncinate lobe of the pancreatic head, when this part of the pancreas is resected with a PDAC. Again, these aggregates which can have a NET-like appearance reveal their normal islet cell type composition when tested for pancreatic polypeptide [79].

## Conclusion

The diagnostic and therapeutic management of PanNENs has very much improved during the last two decades in which NET centers have emerged, supported by societies such as the European Neuroendocrine Tumor Society. This development was accompanied and supported by the steady improvement of classifications, diagnostic criteria, and prognostic assessments. The basis of all, the morphology, is still the starting point of diagnosis. The diagnostic criteria for assessing PanNEN histology have been refined and expanded at the same time in an attempt to capture the tumor´s individuality, to which our attention is more and more directed. This path is continued by the use of biomarker immunohistology that reveals further properties of tumor cells which help to improve classification, precisely record proliferative activity, specify therapeutic approaches, and determine function. The final approach to the diagnosis is the genetic evaluation of the tumor which is currently increasingly integrated into our diagnostic pathways. The correct application of all the diagnostic criteria should protect us from misdiagnosis and pitfalls. Nevertheless, it is important to know and study the special pitfalls of NENs to avoid their potential confusion with NEN in general and PanNEN in particular.
